# 高效液相色谱-串联质谱法分析游离RNA修饰核苷的研究进展

**DOI:** 10.3724/SP.J.1123.2024.07004

**Published:** 2025-01-08

**Authors:** Lyuye ZHANG, Weibing ZHANG, Hailin WANG

**Affiliations:** 1.中国科学院生态环境研究中心,环境化学与生态毒理学国家重点实验室, 北京 100085; 1. State Key Laboratory of Environmental Chemistry and Ecotoxicology, Research Center for Eco-Environmental Sciences, Chinese Academy of Sciences, Beijing 100085, China; 2.华东理工大学化学与分子工程学院,上海市功能性材料化学重点实验室, 上海 200237; 2. Shanghai Key Laboratory of Functional Materials Chemistry, School of Chemistry & Molecular Engineering, East China University of Science and Technology, Shanghai 200237, China; 3.中国科学院大学, 北京 100049; 3. University of Chinese Academy of Sciences, Beijing 100049, China

**Keywords:** RNA修饰, 代谢物分析, 高效液相色谱-串联质谱, 修饰核苷, RNA modifications, metabolite analysis, high performance liquid chromatography-tandem mass spectrometry (HPLC-MS/MS), methylated nucleosides

## Abstract

核糖核苷酸(RNA)的转录后修饰在基因表达调控中发挥重要作用,而RNA在降解与再合成过程中产生大量游离核苷,包含未修饰的基础核苷和多种类型的修饰核苷。修饰核苷无法被进一步分解,在胞内积累,或在转运蛋白作用下运输到胞外环境,直到被机体代谢,并在过程中发挥信号分子的作用。对生物样本中各级代谢物中修饰核苷的准确鉴定与定量能够为RNA降解和游离修饰核苷排泄调控提供重要信息,协助研究其生物学功能和发现潜在的临床应用。例如,小分子修饰核苷在应激反应和疾病状态下作为信号分子调控机体的应答;病理状态下体液和组织中发生特异性变化的修饰核苷可作为生物标志物,为疾病的诊断和治疗提供信息。高效液相色谱-串联质谱(HPLC-MS/MS)技术作为一种高灵敏度、高选择性、快速高效的分析工具,已在游离修饰核苷分析中展现出重要的应用价值。但HPLC-MS/MS准确分析游离修饰核苷仍面临一些挑战,如生物样品成分的复杂性,多种类目标物的化学性质差异,多种目标物的含量呈数量级的差异,以及众多的修饰核苷中同分异构体的干扰等。这些因素使得开发同时定量多种核苷的HPLC-MS/MS方法难度增加。本综述介绍了内源性游离RNA修饰核苷的来源及检测方法,概述了HPLC-MS/MS技术在内源性核苷分析中的样品前处理、色谱分离、质谱检测模式和定量方法开发等方面的研究进展,并综述了游离RNA修饰核苷的生物学功能、调控机制研究和作为疾病潜在分子标志物等的应用。

## Abstract (en)

Post-transcriptional ribonucleic acid (RNA) modifications play crucial roles in regulating gene expression, with both eukaryotic and prokaryotic RNA exhibiting more than 170 distinct and ubiquitous modifications. RNA turnover generates numerous free nucleosides, including unmodified nucleosides and a variety of modified ones. Unlike unmodified nucleosides, modified nucleosides are not further degraded or used in the salvage-synthesis pathway owing to a lack of specific enzymes, which leads to the cytosolic accumulation or cellular efflux of modified nucleosides. These modified nucleosides can act as signaling molecules that regulate downstream pathways once transported to the extracellular space; alternatively, they are metabolized in the bloodstream and excreted in urine. Metabolized modified nucleosides are altered by cellular stress responses and mediate abnormal physiological states. Changes in the urinary and blood levels of modified nucleosides associated with cancer can serve as biomarkers for disease. Therefore, identifying and accurately quantifying nucleosides is vital for understanding RNA degradation and associated patterns of nucleoside metabolism. Such analyses are helpful when studying the biological functions and potential clinical applications of modified nucleosides. In this regard, high performance liquid chromatography-tandem mass spectrometry (HPLC-MS/MS) offers significant advantages in terms of sensitivity, selectivity, and efficiency, and has been widely used to analyze DNA and RNA nucleosides/nucleotides and their analogues. Multiple MS detection patterns and quantification methods have been established to detect nucleosides in biological samples, including cultured cells, urine, blood, and tissue samples. However, the development of an accurate HPLC-MS/MS method faces several challenges. Firstly, the presence of a complex biological matrix that contains macromolecules, small molecules, and salts can interfere with analysis. Salts and co-eluting substances in the extraction solution often affect mass-spectrometric responses for target analytes. Secondly, various nucleosides are present in vastly different abundances, with contents varying by up to four orders of magnitude; hence, accurately quantifying multiple nucleosides in a single assay is challenging. Thirdly, *N*-glycosidic bonds are favorably cleaved in most nucleosides during MS to produce the same characteristic fragment ions, which are often accompanied by nucleobases. This tendency poses challenges for distinguishing structural isomers and mass-analogs of modified nucleosides by MS. Post-transcriptional chemical modifications include methylation, hydroxylation, sulfur/oxygen substitution, and side-chain additions. Developing a unified method for simultaneously screening modified nucleosides is difficult owing to biochemical diversity; consequently, there is a need for advanced HPLC-MS/MS method capable of accurately quantifying such nucleosides. This review summarizes the development and applications of LC-MS technologies for analyzing endogenous nucleosides, covering sample preparation, chromatographic-separation and mass-spectrometric-detection conditions, and the development of quantification methods. Additionally, we discuss applications aimed at detecting and quantifying RNA-derived modified nucleosides in biological samples. The applications of HPLC-MS/MS technology are highlighted, the regulation and function of free modified nucleosides are discussed, and the potential functions of modified nucleosides as disease biomarkers for clinical applications are introduced.

核糖核苷酸(RNA)转录后修饰是指在RNA转录完成后,机体在酶的调控下对RNA分子进行化学修饰的过程。RNA的降解产生基础核苷和无法被进一步降解的修饰核苷,这些修饰核苷作为代谢终产物存在于人体的细胞内外环境、血液和尿液中。近年来随着代谢组学和代谢物研究的发展,对生理和病理状态下代谢通路与调节的认识深入,以及代谢物检测和定量技术的开发和广泛应用,核苷/核苷酸的代谢调控及功能受到了越来越多的关注。研究发现RNA降解产生的修饰核苷具有信号分子功能,其含量在疾病中特异性变化,是潜在的生物标志物。高效液相色谱-串联质谱(HPLC-MS/MS)是针对生物基质内靶向和非靶向代谢物分析的有力工具,HPLC-MS/MS技术已被用于检测细胞系、细胞培养基、组织、血液和尿液中的修饰核苷。使用HPLC-MS/MS技术检测核苷及其类似物的方法被广泛应用于修饰核苷的鉴定、定量及功能研究。

## 1 RNA修饰核苷

### 1.1 RNA修饰核苷的种类

RNA是具有调控基因编码、遗传信息转录、蛋白质合成等多种生物学功能的重要生命大分子,它由基本单元核糖核苷酸构成。每一个核糖核苷酸分子又由核苷和磷酸基团组成。其中,RNA核苷由核糖和含氮碱基构成,RNA核苷在RNA循环、核苷酸代谢及能量合成中发挥关键作用^[1⇓-3]^。除了4种基本核苷(腺苷(rA)、鸟苷(rG)、胞苷(rC)和尿苷(rU))外,真核生物和原核生物中还普遍存在超过170种修饰核苷^[4]^。按照RNA类型分类,几乎所有类型的编码和非编码RNA上都已发现修饰核苷,如编码mRNA以及非编码的tRNA、rRNA、snRNA(small nuclear RNA)等^[5⇓⇓-8]^。按照核苷的含氮碱基种类分类,可以分为嘌呤类修饰核苷,如腺嘌呤的修饰核苷和鸟嘌呤的修饰核苷,以及嘧啶类修饰核苷,如胞嘧啶和尿嘧啶的修饰核苷。按照修饰种类分类,这些转录后化学修饰包括甲基化修饰、羟基化修饰、硫/氧替代或侧链修饰等^[4]^。RNA修饰核苷被报道具有多种功能,比如调控RNA与蛋白质的结合,参与基因的编码、遗传信息转录、蛋白质合成及调控RNA稳定性等。修饰核苷在生命调节的过程中发挥不可或缺的功能^[9⇓⇓-12]^。

### 1.2 代谢RNA修饰核苷的来源

1898年,Krüger等^[13]^在人尿液中发现了7-甲基鸟嘌呤核苷(m^7^G),这是第一种被发现的代谢修饰核苷。1957年,Weissmann等^[14,15]^在人体中检测到其他代谢修饰核苷。随后,Mandel等^[16]^提出甲硫氨酸是尿液中甲基修饰的嘧啶核苷和嘌呤核苷的甲基供体,该假设通过同位素标记的^14^C-甲硫氨酸被验证,人尿液中甲基修饰核苷来源于甲硫氨酸中的甲基^[17]^。Shi等^[18]^使用^13^C-甲硫氨酸标记培养NUGC3细胞,进一步验证胞外修饰的核苷来源于降解的细胞修饰RNA。因此,甲硫氨酸作为RNA甲基化修饰核苷的甲基供体,当RNA(如mRNA、tRNA、rRNA等)降解时其释放的修饰核苷和4种未修饰核苷会成为游离的小分子,并进行下一步代谢。

与4种常规核苷相比,修饰核苷的代谢过程涉及特定的酶,遵循着不同的代谢途径。具体来说,细胞内的常规核苷小分子具有3种代谢方向:(1)未修饰的核苷小分子经过逐步酶解生成代谢终产物,如尿酸、氨基丁酸和*β*-丙氨酸,通过血液和尿液途径排泄至体外;(2)未修饰的核苷通过RNA的“挽救途径”被一系列磷酸激酶磷酸化为三磷酸核苷后重新用于核酸链的合成;(3)未修饰的核苷被平衡型核苷转运蛋白(ENTs)等释放至细胞外空间,作为信号分子与其受体结合,作用于各种机体功能的调节。

与常规核苷不同,随着RNA降解,修饰核苷在细胞内积累。大多数修饰核苷由于缺乏特定的酶而不能进一步代谢,也不能被选择性的磷酸激酶再利用于核酸的补救合成途径^[19]^,修饰核苷与未修饰核苷代谢途径与所涉及的酶如图1所示。Shi等^[18]^研究了修饰核苷从胞内代谢运输到胞外的机制,使用抑制剂和基因敲除技术,通过HPLC-MS/MS检测细胞内外的游离核苷,发现大多数修饰核苷通过平衡核苷转运蛋白1和2(ENT1和ENT2)传输到细胞外空间,如1-甲基腺嘌呤核苷(m^1^A)、*N*^6^-甲基腺嘌呤核苷(m^6^A)、2'-*O*-甲氧基次黄嘌呤核苷(Im)、1-甲基肌苷(m^1^I)、*N*^1^-甲基鸟苷(m^1^G)、*N*^2^-甲基鸟苷(m^2^G)、*N*^2^,*N*^2^-二甲基鸟苷(m^2^_2_G)、3-甲基胞嘧啶核苷(m^3^C)、5-甲基胞嘧啶核苷(m^5^C)、*N*^4^-乙酰基胞嘧啶核苷(ac^4^C)、2'-*O*-甲氧基胞苷(Cm)、2'-*O*-甲氧基尿苷(Um)和假尿嘧啶(Psi),其中ENT1对修饰核苷的偏好性高于ENT2。而少数在N^6^或C^2^位置含有大空间位阻修饰的修饰腺苷(t^6^A、ms^2^t^6^A和ms^2^i^6^A)则不能被ENT1/2的转运结构域运输到胞外,这些修饰核苷胞外转运的机制还有待研究。修饰核苷无法被核糖核酸再利用,其在细胞中的积累造成一定细胞毒性,因此被特定的酶转运到胞外环境。在组织、血液、房水和尿液等生物体多种部位均能检测到游离的修饰核苷。

**图1:**
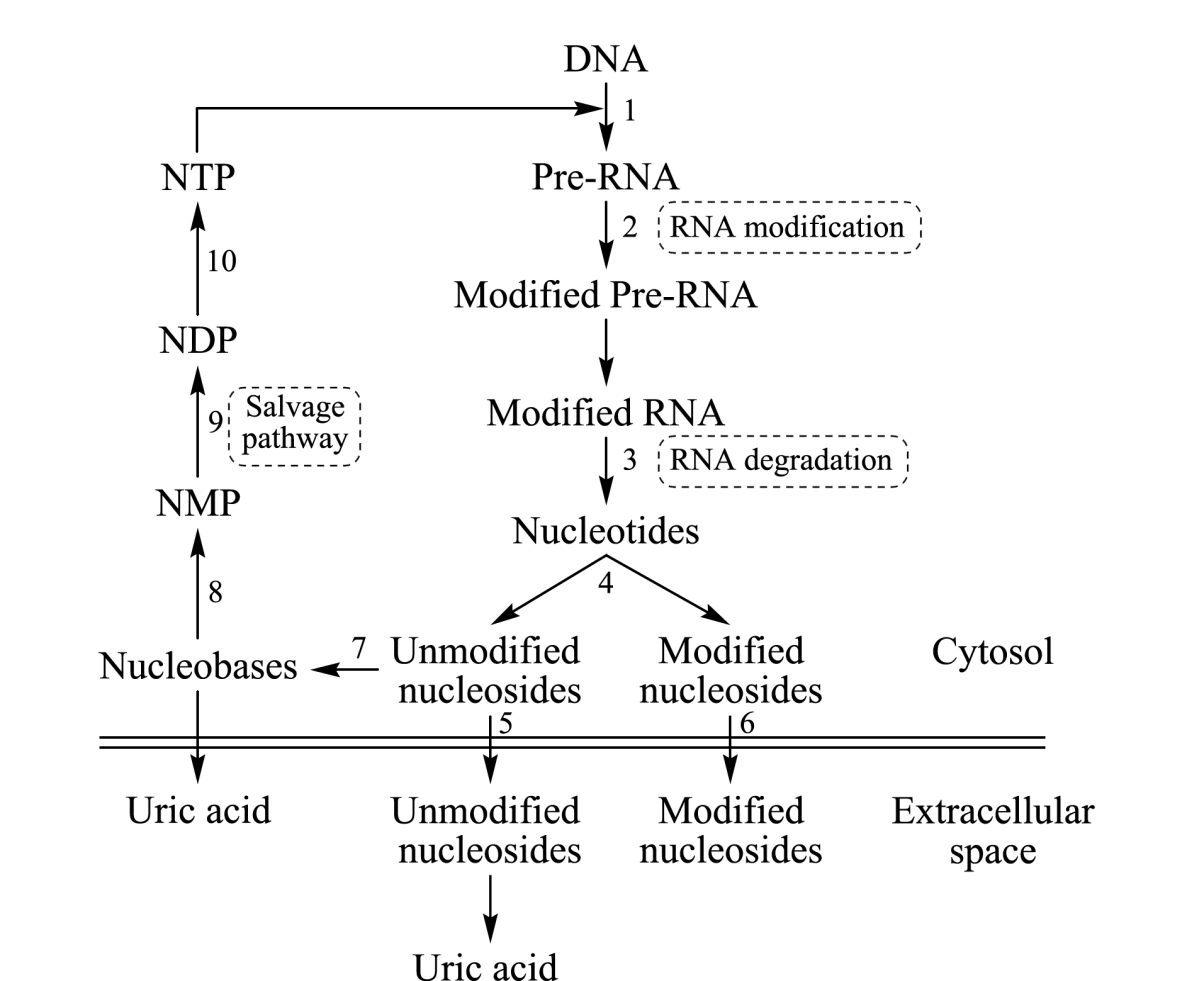
修饰核苷的代谢过程

## 2 RNA修饰核苷的检测方法

### 2.1 HPLC-MS/MS检测RNA修饰核苷

Mandel等^[17]^使用硝酸银与嘌呤核苷形成嘌呤-银沉淀来检测人尿液中的修饰嘌呤核苷。随后几十年,毛细管电泳法(CE)^[21,22]^和液相色谱法(LC)因具有出色的分离能力,与紫外检测器(UV)^[23,24]^或荧光检测器(FLD)联用^[25]^,用于生物样品中游离修饰核苷的检测。

与这些传统检测方法相比,高效液相色谱-串联质谱法,尤其是高效液相色谱-电喷雾电离串联质谱法(HPLC-ESI-MS/MS)已成为目前应用最广泛、研究最全面的方法,应用于RNA和DNA核苷及类似物的鉴定与定量^[26⇓⇓-29]^。质谱检测可以获得更多的结构数据,从而提高对修饰核苷检测和鉴定的能力。HPLC-MS/MS具有高灵敏度,可以实现物质的快速分离和检测,并可以经过稳定同位素稀释法精准定量^[30]^。

HPLC-MS/MS可以靶向检测样品中的目标物,通常采用电喷雾离子源与三重四极杆质谱(QqQ)中的多反应监测模式(MRM),常见分析流程如图2所示。2004年,Dudley等^[31]^使用HPLC-ESI-MS/MS鉴别和检测从尿液中纯化的嘌呤核苷,通过质谱鉴定了两种没有标准品的代谢修饰核苷,并与HPLC-UV进行了比较。由于RNA修饰的核苷修饰基团种类具有多样性,HPLC-UV检测多种游离核苷的分离时间超过1 h^[32]^,而HPLC-MS/MS的质谱定量功能允许不同相对分子质量的物质共流出,分析时间可缩短至几分钟至十几分钟。应用该方法在癌症患者的尿液样品中鉴定出rA、m^1^A、m^1^G、m^2^G、m^2^_2_G、*N*^2^,*N*^2,7^-三甲基鸟苷(m^2,2,7^G)、肌苷(rI)和m^1^I。

**图2:**
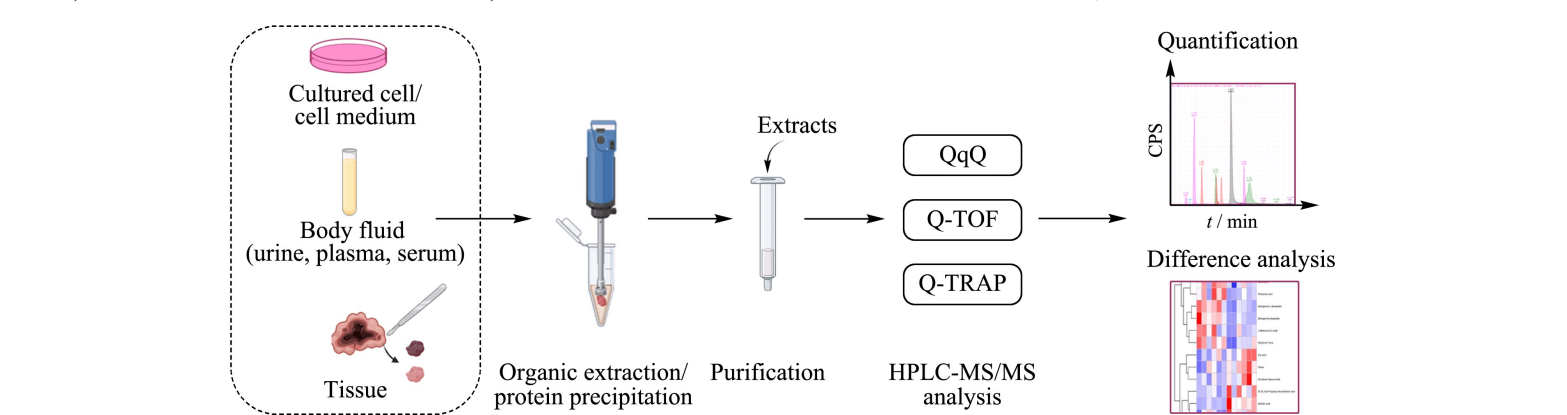
HPLC-MS/MS分析生物基质中修饰核苷的流程图

HPLC-MS/MS也被应用于非靶向筛查样品中的游离核苷,通过对化合物加合离子的高分辨分析实现未知物质的检测与鉴定。Godoy等^[33]^使用四极杆飞行时间质谱(Q-TOF)和四极杆离子阱质谱(Q-TRAP)鉴定未知的修饰核苷,并比较了全扫描(Full scan)、数据依赖的二级质谱采集(Auto MS/MS)、目标离子采集(Target MS/MS)等多种模式下的分析结果。LC-Q-TRAP/MS和LC-Q-TOF/MS方法对人尿液和血清样品中修饰核苷的结构表征均具有较好的灵敏度,可用于非靶向核苷的鉴定。

### 2.2 样品检测

#### 2.2.1 样品的提取和纯化

由于生物基质中的复杂大分子、小分子及盐的存在,提取液中的盐和共流出物质通常影响目标修饰核苷的质谱响应。固相萃取法(SPE)能够显著提高LC-MS定量检测修饰核苷的能力。Dudley等^[31,34]^早期研究了纯化尿液中游离核苷应用于质谱检测的方法,系统比较了几种SPE树脂色谱材料,包括Dowex-1(强阴离子交换)、Dowex-50(强阳离子交换)、QAE(formate)Sephadex(强酸性阴离子交换)、QAE(bicarbonate)Sephadex(强碱性阴离子交换)、SP-Sephadex(强酸性阳离子交换,官能团为磺酸丙基)、Chelex-100(强阴离子交换)材料。最终确定在尿液样品经过前处理离心、酸化和中和步骤后,使用顺式二醇亲和柱特异性地对RNA核苷提纯,再使用酸性阳离子交换柱和碱性阴离子交换柱进一步纯化得到优化的效果,标准品的回收率达92.5%,尿样中核苷的加标回收率为72%~82%。顺式二醇亲和材料的特异性是基于碱性环境下硼酸与顺式二醇化合物共价结合形成稳定的五元或六元环酯,而在酸性环境下环酯开环这一特性,实现可控的可逆共价键合反应。顺式二醇亲和材料AFFIGEL-601(BIO-RAD)常应用于选择性地分离富集生物样品中顺式二醇类物质。后来发展出的顺二醛亲和色谱柱Varian PBA柱,与AFFIGEL-601粉末相似,通过硼酸基团与顺式二醇化合物可逆共价结合纯化RNA核苷,使用更加便捷。顺二醇亲和材料仍是目前纯化RNA核苷常用的SPE材料^[33,35,36]^。

2016年,Encarnación等^[37]^开发了一种SPE法对RNA和DNA的衍生物同时提取。不同于顺二醛亲和色谱柱针对RNA修饰,作者使用了一种由3种聚合材料制备的混合吸附剂,该吸附剂在一步操作中可同时提取DNA和RNA的核苷。

Zhang等^[20]^2024年使用HLB柱材料纯化细胞内提取到的游离修饰核苷,检测胞内10种游离修饰核苷。该方法实现了由于细胞提取液基质干扰而不可分离和定量的同分异构体m^1^G和m^2^G的检测,并增强了目标化合物2'-*O*-甲基鸟苷(Gm)的信号响应。目标嘌呤核苷浓缩后用去离子水复溶,然后上样至活化的HLB柱,用去离子水洗涤,然后采用50%甲醇溶液洗脱目标核苷,修饰核苷2'-*O*-甲基腺苷(Am)、m^6^A、*N*^6^,2*'*-O-甲氧基腺嘌呤核苷(m^6^Am)、*N*^6^,*N*^6^-二甲氧基腺嘌呤核苷(m^6^_2_A)、Gm、m^7^G、m^2^G和m^2^_2_G的回收率均高于90%, m^1^A和m^2^G的回收率高于80%。

#### 2.2.2 液相色谱与质谱检测

质谱仪根据*m/z*区分共流出组分并实现定量,对色谱分离的依赖有所减少。但是针对RNA和DNA修饰核苷的色谱分离分析仍然必不可少。因为目前已鉴定的天然修饰核苷种类多(>170种),修饰基团种类有规律,且*β*-糖苷键是质谱分析时最易断裂的共价键并产生含氮碱基和糖苷,因此内源性修饰核苷池天然存在多种同分异构体,质谱分析导致化合物断裂时也容易产生相同*m/z*的子离子。2022年,Lin等^[38]^提出最常用的UHPLC-QqQ基于母离子和子离子共同定性,所以质谱分析误鉴定主要有3个原因:结构异构体混淆、质量类似物干扰、同位素串扰。因此,在质谱解析这些易混淆物质结构前,仍需开发有效的色谱方法实现分离。

分离核苷及类似物常用亲水作用色谱(HILIC)和反相液相色谱(RPLC)。HILIC能够分离极性较强的化合物并适合与电喷雾离子源联用,是分析核苷和核苷酸的常用模式。RPLC更容易实施并提供较好的分离和分析速度,是分析核苷和修饰核苷最常用的方法,被广泛应用于多种样品基质的分析^[39]^。此外,使用改进ESI源的LC-MS平台能够保证反相液相色谱模式下流动相中包含的高比例水溶剂有效蒸发。

Lin等^[38]^使用3种不同的色谱分析柱(BEH-Amide(HILIC)、Discovery HS F5(C18)和Atlantis T3(C18))分离17种核苷类似物标准品,为了分离母离子和子离子加合物*m/z*都相同的同分异构体核苷(例如m^7^G、m^1^G和m^2^G,或m^1^A和m^6^A),以及*m/z*偏差小于1的化合物(例如*m/z*=282/150与*m/z*=283/151的两组游离核苷化合物),提出分离特定的目标核苷时针对物质极性和保留时间选择色谱柱。

液相色谱流动相添加剂不仅能够改善化合物的色谱分离,还会影响质谱响应,理想的流动相应满足以下条件:a)化合物分离度和色谱峰形状良好;b)同分异构体修饰核苷的分离度更高;c)灵敏度及信号响应(信噪比,*S/N*)更高;d)分析时间适合。已有研究比较了多种添加剂类型,包括甲酸、乙酸、乙酸铵、碳酸氢铵、苹果酸等。Godoy等^[33]^对比0.1%和0.05% (v/v)的甲酸和乙酸流动相添加剂,发现0.05% (v/v)乙酸最优。Zhang等^[20]^对比了流动相添加碳酸氢铵、乙酸铵及甲酸的分离效果,发现添加碳酸氢铵后,12种目标修饰和未修饰的嘌呤类核苷质谱响应明显提高,相对于添加0.01%(v/v)甲酸的质谱信号增强了1.7~24.5倍,添加碳酸氢铵对鸟嘌呤类核苷的信号增强显著,rG、Gm和m^1^G的质谱信号增加20倍以上,m^2^_2_G增加10倍以上,m^1^A、m^7^G、m^6^_2_A和m^2^G的响应增加了5倍以上;还通过优化碳酸氢铵的浓度改善了同分异构体m^1^G和m^2^G的分离效果,如图3所示。

**图3:**
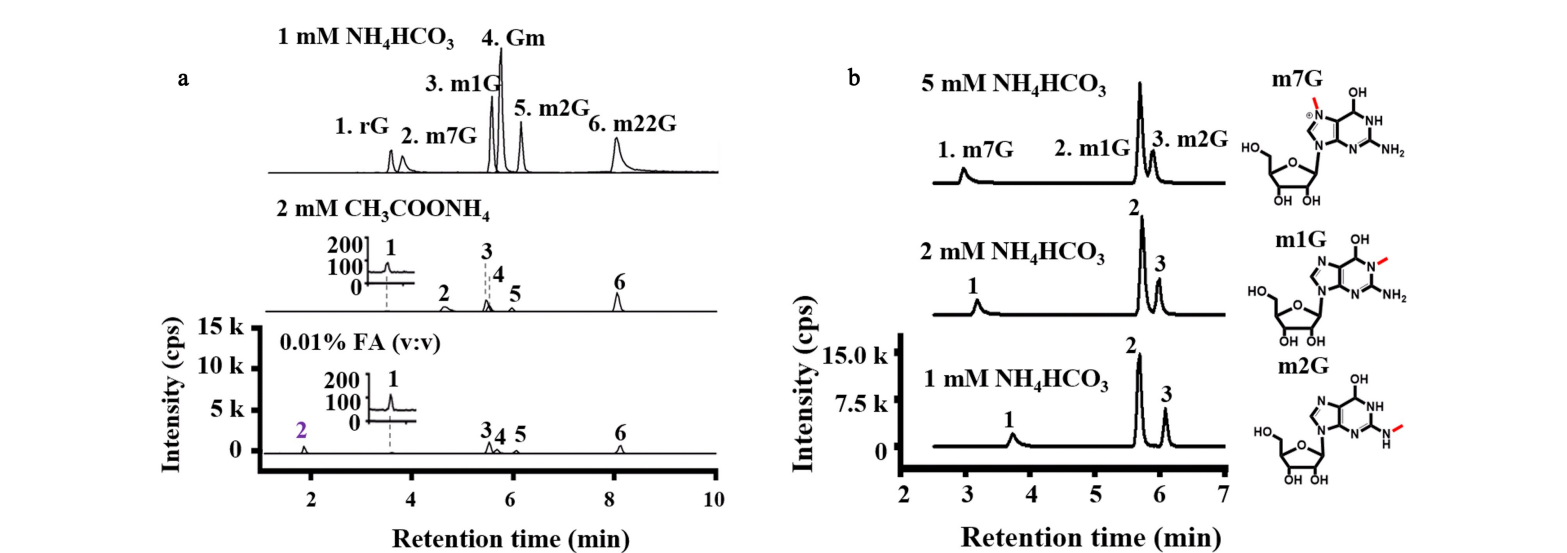
优化流动相添加剂的种类和浓度以增强RNA修饰核苷的(a)信号响应和(b)同分异构体分离

质谱响应增强可归因于在ESI电离过程中碳酸氢铵促进质子向核苷的转移效率。已有研究报道了几种碳酸氢铵增强质谱信号的机制,包括检测修饰的脱氧核苷^[40,41]^、核苷类似物^[42]^及DNA加合物^[43]^。Yin等^[42]^在UHPLC-MS/MS检测DNA胞嘧啶修饰时,使用碳酸氢铵作为流动相添加剂增强响应信号,提出碳酸氢铵通过促进电喷雾离子化过程中目标物的质子化并抑制金属-化合物复合物的形成来增强DNA加合物的质谱信号。碳酸氢铵的 ${\mathrm{NH}}_{4}^{+}$ 可能与核苷类似物形成离子复合物,在溶剂蒸发过程中去氨化为质子化合物。同时, ${\mathrm{HCO}}_{3}^{-}$ 可能形成碳酸,并在热分解中生成二氧化碳和水,该过程有利于核苷类似物的质子化。Paulikat等^[43]^进行了经典分子动力学和量子力学自由能计算,以展示在使用碳酸氢铵作为添加剂时,质谱仪电喷雾过程中质子从 ${\mathrm{NH}}_{4}^{+}$ 转移到DNA七聚体的过程。另一项由Hedges等^[44]^进行的工作比较了碳酸氢铵和乙酸铵在蛋白质质谱分析中的应用,显示碳酸氢铵改善了pH稳定性,因此在电离过程中提供了更稳定的H^+^浓度状态,这可能有助于质子转移效率。

### 2.3 定量方式

质谱检测内源性物质缺少真实空白基质时,定量准确性受到以下因素影响:(1)复杂基质中存在影响MS响应的物质而降低方法重现性;(2)使用标准曲线定量忽视了实际样本的基质效应;(3)无法进行真实的基质效应评价与校正;(4)基质的影响提高了定量限。通常通过适当的样品制备过程和校准方式避免基质效应的影响。纯溶液制备的校准曲线和质量控制(QC)样品简便快捷,但忽略了实际样本的基质效应,导致MS检测响应偏差。使用基质加标法可以适当改善偏差,例如早期使用合成的生物基质来模拟尿液基质并在合成基质中加入标准溶液制作标准曲线实现定量^[33]^。然而细胞、组织或体液的提取物中核苷种类众多,其含量水平的差异较大,如修饰核苷与未修饰核苷含量差异达到4个数量级以上,因此该方法在实际操作时存在困难。

目前,稳定同位素稀释质谱(IDMS)是满足国际计量可溯源性标准的定量方法,使用同位素标记的目标物作为内标与样品同时检测,能够实现基于同位素稀释的定量,并规避上述基质效应带来的影响,对于研究不同RNA修饰核苷功能及其作为疾病生物标志物的精确定量十分必要。同位素内标是物质的部分元素被同位素取代的类似物,如^2^H、^15^N、^13^C标记,可作为特定核苷的内标在相同的保留时间出峰及在同样的运行时间内进行准确定量,改善方法准确性与灵敏度。然而,商品化的同位素标记的修饰核苷种类有限,其他同位素标记修饰核苷需经过化学或生物合成^[45]^,限制了同位素稀释质谱法的广泛使用。商品化的RNA核苷同位素标记物包括标记的rA、rG、rC、m^6^A、m^5^C等。同位素标记的天然和修饰的核苷酸/核苷可以通过使用模式生物并在培养基中加入同位素标记的物质结合制备色谱进行制备。如Quinlivan等^[46]^用^15^ ${\mathrm{NH}}_{4}^{+}$ 标记的培养基培养大肠杆菌,并从中获得^15^*N*-脱氧胞苷和^15^*N*-5-甲基-2'-脱氧胞苷。2014年,Kellner等^[47]^以^13^C-葡萄糖为唯一碳源培养细菌,制备了细菌RNA的稳定同位素标记内标混合物,与通常使用的单独可定量的修饰核苷内标不同,稳定同位素标记内标由多种修饰核苷混合物组成,涵盖了大肠杆菌所有可检测的RNA修饰。研究者使用该混合同位素标记内标经过外部标准曲线校准和样品加标来实现绝对及相对定量。

## 3 应用

### 3.1 小分子修饰核苷的调控与功能

如前所述,已有研究报道了部分修饰核苷的胞外转运蛋白,但另一些大体积修饰核苷转运到胞外环境的机制尚不明确,胞内修饰核苷的积累或外排的调控机制有待深入研究。外排后,修饰核苷可作为内源性信号分子引发生理状态改变。2021年,Ogawa等^[48]^使用C18色谱柱,UHPLC-ESI-QqQ检测了修饰核苷对于腺苷A1、A2、A3受体的结合亲和力,发现m6A是一种内源性腺苷A3受体配体,与A3受体的结合力高于未修饰的腺苷。而几种细胞毒性刺激可以通过溶酶体介导的RNA分解代谢诱导rRNA和mRNA降解,使m^6^A释放到胞外环境,进而促进机体过敏和炎症反应。2021年,Shi等^[18]^检测了细胞上清液和胞质中26种修饰核苷与未修饰核苷,筛选了能够诱导胃癌细胞NUGC3细胞自噬的修饰核苷,发现其中m^1^G诱导细胞自噬的标志物水平上升(*P*< 0.01),说明修饰核苷有可能是潜在的诱导细胞自噬的内源性分子。

Lyu等^[49]^用细胞毒性试剂处理病人样本中分离培养的胶质母细胞瘤细胞,并检测胞内释放的游离核苷。通过提取细胞和培养基中的游离m^6^A和rA,HPLC-MS/MS进行测定发现,使用过氧化氢和脂多糖处理细胞可增加胶质母细胞瘤细胞释放到胞外的m6A。而未修饰的rA的胞外水平减少。缺氧的培养环境可减少胶质母细胞瘤细胞释放到胞外的m^6^A。对于在不同生理和病理条件下RNA降解产生的游离核苷的含量变化、代表的意义以及在体内发挥的功能,仍需继续探索。

Yakita等^[50]^研究了在培养基中加入*N*^6^-异戊烯基腺苷(i^6^A)诱导细胞死亡的分子机制。发现添加到细胞培养基中的i6A会部分掺入到细胞RNA中,主要是18S和28S rRNA,并且抑制RNA聚合酶Ⅰ(PolⅠ)能够抑制这种掺入。而失活的i6A修饰酶TRIT1不影响i6A的掺入。使用不同浓度的i6A培养5-氟尿嘧啶(5-FU)耐药的人口腔鳞状细胞癌细胞系FR2-SAS和5-FU敏感的癌细胞系SAS细胞,发现i6A培养细胞降低了细胞蛋白质合成速率,增加了细胞内蛋白质聚集,并且i6A对5-FU耐药性癌细胞的作用比5-FU敏感的癌细胞更加明显,显示了i^6^A作为对抗5-FU耐药癌细胞的候选化疗药物的可能性。

### 3.2 RNA修饰作为潜在疾病标志物

近十年来,对癌症代谢改变的研究已成为癌症研究的重点之一^[51⇓-53]^。RNA的周转受到多种因素调控,疾病状态下,RNA的周转模式发生改变^[54]^,如tRNA在癌症病人体内周转速度增加^[18,47]^。1977年,Lakings等^[55]^在正常猴子和*N*-亚硝基二乙胺诱导的肝细胞癌猴子中发现,细胞增殖引起RNA周转的显著改变和RNA甲基转移酶活性的增加,癌症个体的生物体液中修饰核苷的排泄显著改变。

游离RNA修饰核苷在癌症中的异常代谢及含量改变已被广泛报道,并作为潜在疾病标志物。研究开发了适用于检测不同生物样本中核苷的方法,如细胞系^[36,56,57]^、组织^[58]^、血液^[59]^和尿液^[24,35,60⇓⇓-63]^。Willmann课题组^[36]^开发了二维LC-MS方法,优化了2D LC-MS的色谱条件,并应用于乳腺癌细胞培养基中修饰核苷的检测。对比乳腺癌细胞系MCF-7和乳腺上皮细胞系MCF-10A的外排核苷及代谢核苷类似物发现,m^5^C、m^1^I、m^6^A、ac^4^C和*N*^6^-苏氨酰氨基甲酰腺苷酸(m6t6A)仅能够在乳腺癌细胞系MCF-7的培养基中检测到,rI和rA仅能够在非癌细胞系MCF-10A培养基中检测到。说明游离核苷的代谢在不同细胞系,包括癌症与非癌细胞之间,可能存在不同的模式。Willmann等^[56]^研究了不同亚型的乳腺癌细胞系和MCF-10A的胞外分泌小分子。细胞培养上清的预纯化采用顺式二醇特异性亲和纯化,应用反相色谱与三重四极杆质谱对样品进行分析。从细胞培养基中检测到23种化合物,包括嘌呤代谢途径中小分子化合物。观察到乳腺癌细胞系与非癌细胞MCF-10A之间,以及不同乳腺癌细胞系之间的RNA代谢的相关通路的代谢模式具有明显的差异,显示了修饰的核苷具有亚型特异性生物标志物的潜力。

已有大量研究报道了癌症患者尿液中修饰核苷含量改变。尿液中核苷类代谢物的水平是研究RNA周转^[64]^和蛋白质代谢的有效参数^[65]^,尿液代谢物也可以作为临床监测的肿瘤标志物,具有采样无创性及操作便利的优势。2006年,Seidel等^[24]^检测了不同类型和进程的肿瘤患者(*n*=68)以及健康对照者(*n*=41)的尿液样本,发现7种游离核苷类似物(rC、伪尿苷(Psi)、2-吡啶酮-5-甲酰胺-*N*^1^-呋喃糖苷(PCNR)、m^2^_2_G、m^1^G、m^2^G和m^1^A)与肌酐的比值在肿瘤患者中显著升高。使用这7种具有显著含量差异的核苷中3种(或4种)诊断肿瘤的敏感性为54%(77%),特异性可达到86%(98%)。进一步与使用癌胚抗原、癌抗原15-3和组织多肽抗原的结果比较表明尿液中游离修饰核苷可作为有效的临床肿瘤标志物。

在不同癌症类型中均发现了修饰核苷的代谢差异。Struck等^[35]^研究了泌尿生殖系统肿瘤患者的尿样中10种修饰核苷的含量差异,结果显示癌症患者(*n*=68)和健康对照者(*n*=61)的尿液样本中5种核苷(m^6^A、I、m^2^G、3-甲基尿苷(m^3^U)和m^2^_2_G)水平之间存在显著差异(*P*<0.05)。而其他的游离核苷(C、Psi、3-甲基胞苷(m^3^C)、5-甲基尿苷(m^5^U)及Xanthosine(X))不具有显著性差异。

Guo等^[62]^开发了基于Fe_3_O_4_/石墨烯的磁分散固相萃取富集法,流动相中添加苹果酸改善目标修饰核苷的分离并提高其质谱检测的灵敏度。开发亲水作用液相色谱-质谱联用方法定量人体尿液样品中5种嘌呤类修饰核苷/脱氧核苷(*N*^6^-甲基-2'-脱氧腺苷(6mA)、m^6^A、Am、m^6^Am和m^6^_2_A)。该研究准确定量了胃癌和结直肠癌患者以及健康对照者的尿液样本中目标修饰腺嘌呤核苷的含量,发现胃癌和结直肠癌患者尿液中6mA和Am水平相对于健康对照组显著降低(胃癌患者与健康对照尿液中Am含量差异*P*<0.05,其余*P*<0.01);胃癌患者尿液m^6^A水平显著降低(*P*<0.01),而结直肠癌患者尿液m^6^A水平对于健康对照组升高(*P*<0.05)。揭示了尿液中甲基化腺嘌呤核苷的水平与胃癌和结直肠癌的发生之间的关联。

2022年,Fang等^[63]^使用亲水作用液相色谱-串联质谱结合稳定同位素稀释法,用于定量检测健康对照和乳腺癌患者尿液样本中10种修饰核苷,发现m^6^A、Am、m^1^A、m^6^Am、m^1^G、Gm、m^5^C和Cm在早期乳腺癌患者尿液中均有所下降。但是,与早期乳腺癌患者相比,局部晚期乳腺癌患者尿液中2'-*O*-甲基化核苷水平升高。

此外,2018年,Guo等^[58]^开发了基质辅助激光解吸电离质谱成像(MALDI-MS)方法检测核苷在癌症组织中的含量。胃癌的诊断通常基于活检和组织学证实,主要治疗手段是手术切除,因此,研究者使用MALDI-MS检测小分子代谢物在胃癌组织(*n*=19)中的分布,发现相对于癌旁区域,癌组织区的核苷酸,如单磷酸尿苷(UMP)、单磷酸腺苷(AMP)和单磷酸鸟苷(GMP)显著升高,核苷(肌苷、鸟苷和尿苷)显著降低。19对组织样本中核苷与其对应的核苷单磷酸的水平比值在癌症和癌旁组织间存在显著差异,核苷与其单磷酸的比值可以作为癌组织病理诊断的指标。

## 4 结论与展望

本文对生物样本中游离RNA修饰核苷的HPLC-MS/MS定性与定量分析方法进展进行了综述,主要阐述了内源性RNA修饰核苷的来源,HPLC-MS/MS检测的模式、色谱条件优化、定量方法发展,以及HPLC-MS/MS检测应用于RNA修饰核苷的调控和作用机制研究以及在疾病的分子标志物筛选中的进展。目前所开发的HPLC-MS/MS定性与定量方法已被广泛应用于DNA核苷、RNA核苷的分析,未来研究的重点是开发能够应用于同时分析复杂生物样本中多种修饰核苷目标物的高灵敏筛查方法,以及开发同位素稀释质谱法以实现特定目标修饰核苷的精确定量。在前处理方面,需对动态的代谢物进行即时快速定量以反应响应生理或病理状态下的分子信息。

游离修饰核苷含量在多种癌症,多类型的生物样本(细胞、细胞外泌培养基、尿液、组织等)中显示了显著差异,并在早期癌症诊断、癌症分型等临床诊疗中作为潜在分子标志物。但是,修饰核苷的含量变化及代谢模式在不同癌症种类及分期中有所不同。目前对RNA修饰核苷的代谢模式和生物学功能的探索仍处在早期阶段,例如,细胞对胞内累积的修饰核苷和其外排的调控,以及对未检测到的多种核苷的运输机制的了解仍为冰山一角,它们在生理和病理环境中的功能和调节过程在少数的模型中初见端倪,为系统深入地研究RNA修饰核苷作为信号分子的潜在能力提供新思路。因此,开发量化修饰核苷的方法,并结合其他组学和生物学实验深入研究修饰核苷代谢及其在健康和疾病中的差异与生物机制是未来的需求方向。
